# Incidence of Hypothyroidism and Thyroid Function Monitoring After Immune Checkpoint Inhibitor Therapy Completion for Lung Cancer: A Nationwide Analysis of a Japanese Claims Database

**DOI:** 10.3390/curroncol32100558

**Published:** 2025-10-04

**Authors:** Hiroaki Ohta, Hinako Tsugane, Takeo Yasu

**Affiliations:** Education and Research Unit for Comprehensive Clinical Pharmacy, Department of Medicinal Therapy Research, Meiji Pharmaceutical University, Tokyo 204-8588, Japan

**Keywords:** immune checkpoint inhibitors (ICIs), hypothyroidism, thyroid function monitoring, immune-related adverse events (irAEs), lung cancer, claims database

## Abstract

**Simple Summary:**

Immune checkpoint inhibitors (ICIs) have significantly advanced lung cancer treatment but can lead to immune-related side effects, including thyroid dysfunction. While prior research has focused on thyroid issues during treatment, little is known about thyroid issues emerging post therapy. In this nationwide Japanese study, 4% of patients developed hypothyroidism requiring thyroid hormone therapy after ICI discontinuation, with a median onset of 67 days. Although 73.7% of patients underwent thyroid function testing after treatment, the most common (modal) timing of testing was just 21 days post-discontinuation—well before the typical onset of hypothyroidism. This suggests that current monitoring practices may not fully capture late-onset cases. ICI combined with bevacizumab and preexisting myasthenia gravis increased the risk, while long-term steroid use lowered the risk. These findings emphasize the need to extend thyroid monitoring beyond the initial few weeks after ICI therapy, potentially improving timely diagnosis and patient care in future clinical practice.

**Abstract:**

Immune checkpoint inhibitors (ICIs) improve lung cancer prognosis but are associated with immune-related adverse events, most commonly thyroid dysfunction. While prior studies and guidelines have focused on thyroid dysfunction during ICI therapy, data on hypothyroidism and its monitoring after ICI therapy remain limited. We aimed to investigate hypothyroidism incidence and implementation of thyroid function monitoring after ICI therapy completion in patients with lung cancer. We conducted a retrospective observational study using the DeSC claims database of approximately 12 million individuals in Japan. Patients with lung cancer who received ICI therapy between April 2014 and August 2023 were included; those with a history of thyroid hormone replacement or insufficient follow-up were excluded. Among 6883 eligible patients, 277 (4.0%) developed hypothyroidism requiring hormone replacement post-ICI therapy completion (median onset, 67.0 d). Risk factors included ICI plus bevacizumab therapy and a history of myasthenia gravis, while steroid use for ≥28 d during ICI therapy lowered the risk. Post-ICI therapy completion thyroid monitoring was performed in 73.7% of patients, with test date distribution showing a median of 126.0 d and mode of 21.0 d. Hypothyroidism was frequently found to develop within 2 months post-ICI therapy completion, highlighting the need for continued thyroid monitoring and prospective studies to establish optimal surveillance strategies.

## 1. Introduction

Immune checkpoint inhibitors (ICIs) have revolutionized lung cancer treatment [1,2,3,4]. However, ICIs can cause immune-related adverse events (irAEs), with representative manifestations including dermatologic, gastrointestinal, and thyroid disorders [5,6]. Among these, thyroid dysfunction may be asymptomatic or present with nonspecific symptoms such as fatigue, weight loss, or constipation, making thyroid-stimulating hormone (TSH), free triiodothyronine (FT3), and free thyroxine (FT4) function monitoring essential for appropriate management [7,8]. Hypothyroidism is the most common thyroid dysfunction associated with ICIs. The incidence of hypothyroidism is 2.5–3.8% during ICI therapy with anti-cytotoxic T-lymphocyte antigen 4 (CTLA-4) antibodies, 3.9–8.5% with anti-programmed death receptor 1 (PD-1)/programmed death-ligand 1 antibodies, and 10.2–16.4% with combination therapy [9]. Hypothyroidism typically develops within 8–12 weeks of ICI treatment initiation [9]. Hypothyroidism has also been reported after ICI therapy completion [10]. However, prior studies on ICI-related hypothyroidism [9,11,12] and guidelines on thyroid function monitoring [7,8] primarily focus on the period during ICI therapy, resulting in limited knowledge on hypothyroidism incidence and implementation of thyroid function monitoring post-ICI therapy completion. Therefore, we conducted a retrospective study using the DeSC claims database with the aim to investigate hypothyroidism incidence and the implementation of thyroid function monitoring after ICI therapy completion in patients with lung cancer. Our research provides valuable findings informing oncology practice and patient management regarding hypothyroidism following ICI therapy completion. 

## 2. Materials and Methods

### 2.1. Study Design and Data Source

This retrospective study investigated hypothyroidism incidence after ICI therapy completion and the implementation status of thyroid function test monitoring across Japan. Data were obtained from the DeSC database, a commercial administrative claims repository provided by DeSC Healthcare Inc. (Tokyo, Japan; https://desc-hc.co.jp/). As of October 2022, the database included approximately 12 million insured individuals and integrated information from three major health insurance schemes under Japan’s universal healthcare system: Kenpo (for employees of large corporations), Kokuho (non-employees, retirees, and their dependents), and the Koki Koreisha Iryo Seido (individuals ≥ 75 years old). Collectively, these schemes cover approximately 10% of the Japanese population and provide a comprehensive view of nationwide healthcare utilization. The study was conducted in accordance with the principles of the Declaration of Helsinki and approved by the Ethics Committee of Meiji Pharmaceutical University (No. 202514).

### 2.2. Study Population

**Figure 1:**
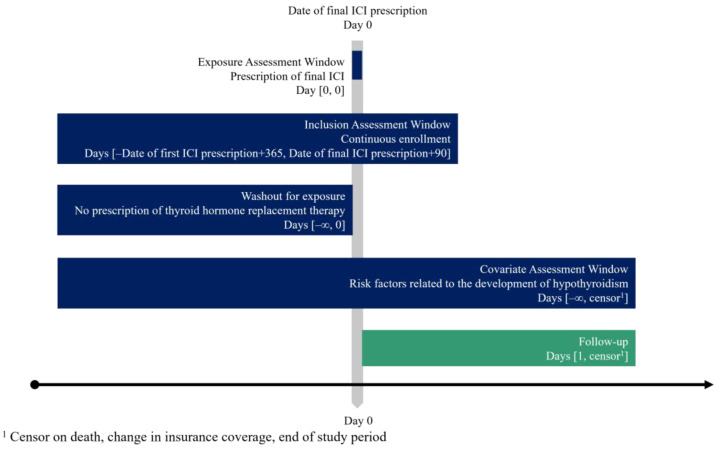
Study design and time windows for inclusion, covariate assessment, and follow-up. ICI, immune checkpoint inhibitor.

Among 1,838,927 patients with confirmed cancer diagnoses (based on International Classification of Diseases, 10th Revision [ICD-10] codes beginning with “C”, excluding suspected cases) between April 2014 and August 2023, patients with lung cancer (ICD-10 codes: C33 and C34) prescribed ICIs (Anatomical Therapeutic Chemical [ATC] codes: L01FF01, L01FF02, L01FF03, L01FF05, L01FX04, and L01FX20) were identified. The index date was defined as the date of the final ICI prescription. Patients were excluded if data were available for <365 d from the first ICI prescription or <90 d from the final ICI prescription (Figure 1). Additionally, patients with a history of thyroid hormone replacement therapy (ATC code: H03AA) before the final ICI prescription were excluded.

### 2.3. Outcomes

#### 2.3.1. Hypothyroidism After ICI Therapy Completion

The incidence of hypothyroidism requiring hormone replacement after ICI therapy completion and its associated risk factors were evaluated. Hypothyroidism was defined as the presence of an ICD-10 diagnosis code for hypothyroidism (E02 and E03) and a prescription for thyroid hormone replacement therapy (ATC code: H03AA) after the final ICI prescription. This definition corresponds to Grade ≥ 2 hypothyroidism according to the Common Terminology Criteria for Adverse Events (CTCAE), version 5.0 [8,13].

#### 2.3.2. Thyroid Function Monitoring After ICI Therapy Completion

The implementation status of thyroid function monitoring was assessed after ICI therapy completion. Monitoring was defined as performing any of the following tests: TSH, FT3, FT4, TSH receptor antibody, or thyroid-stimulating antibody.

### 2.4. Statistical Analyses

Continuous variables are expressed as medians with interquartile ranges (IQRs), and categorical variables are summarized as numbers and percentages. The Mann–Whitney U and Fisher’s exact tests were used to compare patient characteristics. To identify factors associated with hypothyroidism incidence after ICI therapy completion, univariable and multivariable logistic regression analyses were performed with the following covariates: sex, age (≥75 years), ICI regimen type, ICI type, presence of autoimmune disease at ICI therapy initiation (defined as the first ICI prescription), and steroid use ≥28 d during ICI therapy. ICI therapy duration was defined as the time from the date of the first ICI prescription to the date of the final ICI prescription. Both the number of ICI cycles and ICI therapy duration were dichotomized using the median value as the cutoff. Variables with *p* < 0.1 in univariable analysis were included in the multivariable model. All statistical analyses were conducted using EZR (version 2.9–1; Saitama Medical Center, Jichi Medical University, Saitama, Japan) [14]. A two-sided *p* < 0.05 was considered statistically significant.

## 3. Results

### 3.1. Patient Characteristics

**Figure 2:**
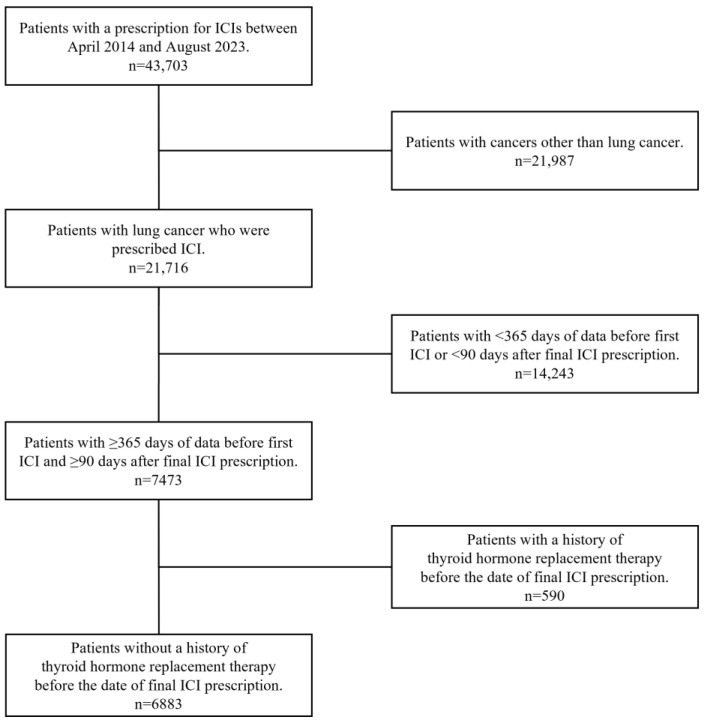
Flowchart of patients included in the study.

Among 43,703 patients prescribed ICIs and registered in the DeSC database between April 2014 and August 2023, 21,716 were diagnosed with lung cancer. Of these, 6883 met the inclusion criteria, requiring adequate observation time to confirm continuous insurance enrollment and thyroid hormone replacement therapy history before the final ICI prescription (Figure 2). Among eligible patients, 5290 (76.9%) were male. The median age of the patients was 74.0 years (IQR, 69.0–79.0). ICI monotherapy was the most common regimen (5181 patients, 75.3%), followed by combination therapy with cytotoxic anticancer agents. By ICI type, pembrolizumab was most frequently prescribed (3118 patients, 45.3%). The median number of ICI cycles was 5.0 (IQR, 3.0–10.0), and the median ICI therapy duration was 105.0 d (IQR, 42.0–226.0). At ICI therapy initiation, autoimmune disease history included 265 patients (3.9%) with rheumatoid arthritis, 23 (0.3%) with systemic lupus erythematosus, 20 (0.3%) with type 1 diabetes, 12 (0.2%) with ulcerative colitis, 6 (0.1%) with myasthenia gravis, and 2 (0.03%) with Crohn’s disease. During ICI therapy, 761 patients (11.1%) received oral or injectable steroids for ≥28 d (Table 1).

### 3.2. Incidence of Hypothyroidism After ICI Therapy Completion

A total of 277 patients (4.0%) developed hypothyroidism requiring thyroid hormone replacement therapy after ICI therapy completion, corresponding to CTCAE Grade ≥ 2. The median time to onset was 67.0 d (IQR, 28.0–141.5) after ICI therapy completion. Hypothyroidism risk factors included ICI plus bevacizumab therapy (odds ratio [OR], 2.17; 95% confidence interval [CI], 1.07–4.39; *p* = 0.031) and a history of myasthenia gravis at ICI initiation (OR, 12.70; 95% CI, 2.31–70.00; *p* = 0.003). Conversely, patients who received oral or injectable steroids for ≥28 d during ICI therapy had a reduced risk of developing hypothyroidism after ICI therapy completion (OR, 0.52; 95% CI, 0.31–0.87; *p* = 0.013) (Table 2).

### 3.3. Thyroid Function Monitoring After ICI Therapy Completion

**Figure 3:**
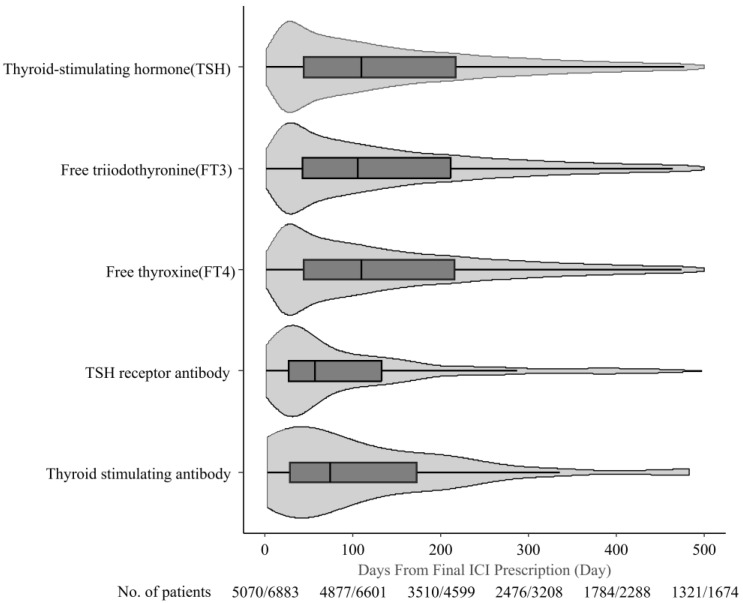
Time from final ICI prescription to thyroid function test monitoring.

Among the eligible patients, 5070 (73.7%) underwent at least one guideline-recommended thyroid function test (TSH, FT3, or FT4) after ICI therapy completion (Table 1). Additionally, 232 (3.4%) and 44 patients (0.6%) underwent TSH receptor antibody and thyroid-stimulating antibody testing, respectively. The median (IQR) number of days from post ICI therapy completion to testing was 126.0 d (IQR, 50.0–273.0) for TSH, 126.0 d (IQR, 49.0–273.0) for FT3, 126.0 d (IQR, 49.0–273.0) for FT4, 60.0 d (IQR, 27.0–154.0) for TSH receptor antibody, and 75.0 d (IQR, 28.8–183.5) for thyroid-stimulating antibody. The mode was 21.0 d for TSH, FT3, FT4, and TSH receptor antibody, and 42.0 d for thyroid-stimulating antibody (Table 3, Figure 3).

## 4. Discussion

This study is the first retrospective observational analysis in Japan to investigate the incidence, timing, and monitoring of hypothyroidism after ICI therapy completion in patients with lung cancer who had not developed Grade ≥ 2 hypothyroidism during treatment. Using a nationwide claims database, we also evaluated patient factors associated with hypothyroidism onset after ICI therapy completion.

Among 6883 eligible patients, 277 (4.0%) developed new-onset Grade ≥ 2 hypothyroidism after ICI therapy completion, with a median time to onset of 67.0 d. In contrast, hypothyroidism incidence during ICI therapy is approximately 6.0–10.0% in patients with lung cancer [6,15]. Although the frequency observed in this study was lower than that during treatment, prior studies included Grade 1 cases. As most ICI-related hypothyroidism cases are Grade 1–2, and Grade ≥ 3 cases are rare [16], our findings suggest a post-ICI therapy rate comparable to that during therapy. To date, the only other study that specifically assessed post-ICI Grade ≥ 2 hypothyroidism was a U.S. single-center study in patients with non-small-cell lung cancer, which found a 2.5% incidence [17], comparable to our findings. Notably, in that study, median time to thyroid hormone initiation after ICI therapy completion was 126 d, approximately 2 months later than that in our cohort. Furthermore, thyroid function testing after ICI therapy completion was performed in 73.7% of Japanese patients compared with only 34.0% in the U.S. study [17], suggesting that structured monitoring in Japan enabled earlier detection. However, 26.3% of patients in our cohort did not undergo post-ICI thyroid testing, indicating possible underestimation of hypothyroidism incidence. Another possible source of underestimation is our focus on Grade ≥ 2 hypothyroidism, defined by ICD-10 diagnosis and thyroid hormone prescription, which may exclude asymptomatic or subclinical (Grade 1) cases. According to the ESMO guidelines [8], Grade 1 hypothyroidism includes cases that require only observation. While we prioritized clinically significant cases to enhance relevance to therapeutic decision-making, patients with Grade 1 or subclinical hypothyroidism may also require careful follow-up, especially after ICI therapy completion. Future studies should aim to incorporate laboratory data to better characterize the full spectrum of thyroid dysfunction.

We identified ICI plus bevacizumab therapy and a history of myasthenia gravis as risk factors for post-ICI hypothyroidism. Bevacizumab, an anti-vascular endothelial growth factor (VEGF) antibody, may contribute to this association via immunomodulatory effects including normalizing tumor vasculature and enhancing the infiltration of CD4+ T-cells, CD8+ T-cells, and mature dendritic cells into the tumor microenvironment [18]. Furthermore, as VEGF suppresses dendritic cell maturation, its inhibition via anti-VEGF antibodies promotes maturation and enhances antitumor immune responses [18]. Thus, combination therapy with ICIs and bevacizumab may augment antitumor efficacy; however, it also increases the risk of hypothyroidism as an irAE after treatment.

A history of myasthenia gravis was associated with an increased post-ICI hypothyroidism risk, although only six patients (0.1%) in this study had this condition. To the best of our knowledge, no previous study has reported such an association. Phase III ICI trials have generally excluded patients with preexisting autoimmune diseases because of concerns regarding increased irAEs. Prospective studies evaluating ICI safety in such patients have shown conflicting results: some report higher rates of irAEs in patients with autoimmune disease [19], while others find no significant difference [20,21]. Risk may depend on the type and severity of the autoimmune disease. Therefore, further studies are needed to clarify whether specific autoimmune conditions, such as myasthenia gravis, predispose patients to post-ICI irAEs.

Steroid use for ≥28 d during ICI therapy was associated with significantly reduced risk of hypothyroidism post-ICI therapy completion. This may have resulted from corticosteroid properties, including inhibition of CD4+ and CD8+ T-cell proliferation, suppression of cytokine production (e.g., IFN-γ, IL-2), impairment of dendritic cell differentiation and maturation, and induction of apoptosis, all of which may attenuate ICI-driven thyroid autoimmunity [22]. However, the database did not specify steroid use indications, which may have included preexisting autoimmune diseases or other irAEs. Notably, as irAE management guidelines [7,8] recommend thyroid hormone replacement for hypothyroidism rather than corticosteroids, the observed association is unlikely to reflect steroid use for thyroid dysfunction itself. Although prolonged steroid use during ICI therapy was associated with a reduced risk of post-therapy hypothyroidism in our analysis, this finding should be interpreted with caution. Steroid use is often indicative of the presence of other irAEs, which may act as confounding factors.

This study is the first to report real-world monitoring practices for thyroid function after ICI therapy completion in Japan. We found that 73.7% of patients underwent at least one thyroid function test after treatment, with testing peaks at approximately 21.0 d, despite guidelines recommending monitoring primarily during treatment [7,8]. However, given that the median onset of hypothyroidism was 67.0 d in our study, continued monitoring for at least 2 months post-ICI therapy may be warranted. Nevertheless, our study was not designed to evaluate whether extended monitoring improves survival or quality of life. Future prospective studies are needed to assess the clinical- and cost-effectiveness of continued thyroid surveillance after ICI therapy completion.

This study has several limitations. First, because claims data were used, confirming whether post-ICI therapy hypothyroidism was directly caused by ICIs was difficult; however, only 9 of the 277 affected patients were prescribed other hypothyroidism-inducing drugs [23]. Second, although we were able to evaluate whether thyroid function tests were performed, laboratory values were unavailable, precluding detailed thyroid function analyses. Third, whether ICI therapy was discontinued because of sufficient therapeutic effect or other reasons such as irAEs was unclear. Therefore, although ICI therapy duration and ICI cycle number were not associated with hypothyroidism incidence after ICI therapy completion in this study, results may vary depending on the reason for treatment discontinuation. Fourth, data of patients who died before hypothyroidism onset may not have been captured. Although we attempted to mitigate immortal time bias by including only patients who survived ≥90 d post-ICI therapy completion, residual bias cannot be excluded. Furthermore, potential confounding factors must be considered when interpreting our findings. Although we excluded patients with preexisting thyroid hormone replacement to reduce baseline bias, unmeasured comorbidities—such as autoimmune diseases, prior cancer therapies, or subclinical thyroid dysfunction—may have influenced the development of hypothyroidism after ICI therapy completion. In addition, although we attempted to minimize immortal time bias, early death during follow-up could have resulted in further underestimation of hypothyroidism incidence. These factors represent potential sources of residual confounding inherent in claims-based observational studies. Therefore, future studies using clinical databases with richer clinical and laboratory data are warranted to more rigorously evaluate these influences.

## 5. Conclusions

This retrospective study, using real-world data in Japan, demonstrated that Grade ≥ 2 hypothyroidism can develop even after ICI therapy completion in patients with lung cancer, with a median onset of 67 d. Combination therapy with bevacizumab was identified as a risk factor, whereas prolonged steroid use during ICI therapy lowered the risk. A history of myasthenia gravis was also a potential risk factor, although cases were few. Although approximately three-quarters of patients underwent thyroid function monitoring post-ICI therapy, our findings indicate that monitoring should continue for at least 2 months after ICI therapy completion. Future studies are needed to establish optimal monitoring strategies and evaluate whether extended surveillance improves outcomes and quality of life.

## Figures and Tables

**Table 1:** Patient characteristics.

	Total, n (%)
	6883
Male sex	5290 (76.9)
Age: median (IQR)	74.0 (69.0–79.0)
Type of ICI regimen	
Monotherapy	5181 (75.3)
Combination	1702 (24.3)
PD-1 + CTLA-4	160 (2.3)
ICI + Cytotoxic anticancer agents	1365 (19.8)
ICI + Bevacizumab	116 (1.7)
ICI + Cytotoxic anticancer agents + Bevacizumab	60 (0.9)
ICI + Lenvatinib	1 (0.01)
ICI-Type	
Pembrolizumab	3118 (45.3)
Nivolumab	1328 (19.3)
Atezolizumab	1416 (20.6)
Durvalumab	1018 (14.8)
Ipilimumab	189 (2.7)
Number of ICI cycles	5.0 (3.0–10.0)
Duration of ICI therapy	105.0 (42.0–226.0)
Thyroid monitoring during ICI therapy	5354 (77.8)
Thyroid monitoring after ICI therapy completion	5070 (73.7)
Thyroid monitoring ≤90 d post-ICI therapy	4481 (65.1)
Autoimmune disease (at the initiation of ICI therapy)	
Systemic lupus erythematosus	23 (0.3)
Crohn’s disease	2 (0.03)
Ulcerative colitis	12 (0.2)
Myasthenia gravis	6 (0.1)
Rheumatoid arthritis	265 (3.9)
Type 1 diabetes	20 (0.3)
Steroid use ≥ 28 d during ICI treatment	761 (11.1)

IQR, interquartile range; PD-1, programmed death receptor 1; CTLA-4, cytotoxic T-lymphocyte antigen 4; ICI, immune checkpoint inhibitor.

**Table 2:** Univariate and multivariate logistic regression models assessing factors affecting odds of developing hypothyroidism after ICI therapy completion.

	Univariate Analysis		Multivariate Analysis	
	Odds Ratio (95% CI)	*p*-Value	Odds Ratio (95% CI)	*p*-Value
Male sex	0.77 (0.59–1.01)	0.062	0.79 (0.60–1.03)	0.079
Age ≥ 75 years	0.96 (0.76–1.23)	0.781		
Type of ICI regimen				
Monotherapy	0.75 (0.57–0.97)	0.028	0.94 (0.69–1.27)	0.665
Combination				
PD-1 + CTLA-4	1.98 (1.08–3.60)	0.026	1.12 (0.24–5.30)	0.889
ICI + Cytotoxic anticancer agents	1.05 (0.78–1.41)	0.751		
ICI + Bevacizumab	2.30 (1.19–4.44)	0.014	2.17 (1.07–4.39)	0.031
ICI + Cytotoxic anticancer agents + Bevacizumab	2.19 (0.87–5.51)	0.096	2.11 (0.81–5.50)	0.126
ICI + Lenvatinib	NA	NA		
ICI-Type				
Pembrolizumab	1.12 (0.88–1.43)	0.354		
Nivolumab	1.09 (0.81–1.46)	0.581		
Atezolizumab	0.87 (0.64–1.18)	0.364		
Durvalumab	0.86 (0.60–1.22)	0.391		
Ipilimumab	1.96 (1.12–3.42)	0.019	1.79 (0.42–7.75)	0.433
Autoimmune disease (at the initiation of ICI therapy)				
Systemic lupus erythematosus	NA	NA		
Crohn’s disease	NA	NA		
Ulcerative colitis	2.17 (0.28–16.90)	0.458		
Myasthenia gravis	12.0 (2.19–65.80)	0.004	12.70 (2.31–70.00)	0.003
Rheumatoid arthritis	1.03 (0.60–1.91)	0.915		
Type 1 diabetes	NA	NA		
Steroid use ≥28 d during ICI treatment	0.48 (0.29–0.80)	0.005	0.52 (0.31–0.87)	0.013

PD-1, programmed death receptor 1; CTLA-4, cytotoxic T-lymphocyte antigen 4; ICI, immune checkpoint inhibitor; CI, confidence interval; NA, not applicable.

**Table 3:** Timing of thyroid function monitoring after ICI therapy completion.

	Thyroid-Stimulating Hormone (TSH)	Free Triiodothyronine (FT3)	Free Thyroxine (FT4)	TSH Receptor Antibody	Thyroid Stimulating Antibody
Median (IQR), d	126.0 (50.0–273.0)	126.0 (49.0–273.0)	126.0 (49.0–273.0)	60.0 (27.0–154.0)	75.0 (28.8–183.5)
Mode, d	21.0	21.0	21.0	21.0	42.0

ICI, immune checkpoint inhibitor; IQR, interquartile range.

## Data Availability

Data presented in this study are available upon request from the corresponding author.

## Abbreviations

The following abbreviations are used in this manuscript:

CI	confidence interval
CTLA-4	cytotoxic T-lymphocyte antigen 4
FT3	free triiodothyronine
FT4	free thyroxine
ICI	immune checkpoint inhibitor
IQR	interquartile range
irAE	immune-related adverse events
OR	odds ratio
PD-1	programmed death receptor 1
TSH	thyroid-stimulating hormone
VEGF	vascular endothelial growth factor
